# Antiseptic Effect of Ps-K18: Mechanism of Its Antibacterial and Anti-Inflammatory Activities

**DOI:** 10.3390/ijms20194895

**Published:** 2019-10-02

**Authors:** Mihee Jang, Jieun Kim, Yujin Choi, JeongKyu Bang, Yangmee Kim

**Affiliations:** 1 Department of Bioscience and Biotechnology, Research Institute for Bioactive-Metabolome Network, Konkuk University, Seoul 05029, Korea; smileday1229@konkuk.ac.kr (M.J.); za3524@konkuk.ac.kr (J.K.); 2 Chuncheon Center, Korea Basic Science Institute, Chuncheon 24341, Korea; cyj4854@gmail.com; 3 Protein Structure Group, Korea Basic Science Institute, Ochang, Cheongju, Chung-Buk 28199, Korea; bangjk@kbsi.re.kr

**Keywords:** pseudin-2, antimicrobial peptide, antisepsis, peptide antibiotics

## Abstract

Recently, bioactive peptides have attracted attention for their therapeutic applications in the pharmaceutical industry. Among them, antimicrobial peptides are candidates for new antibiotic drugs. Since pseudin-2 (Ps), isolated from the skin of the paradoxical frog *Pseudis paradoxa*, shows broad-spectrum antibacterial activity with high cytotoxicity, we previously designed Ps-K18 with a Lys substitution for Leu^18^ in Ps, which showed high antibacterial activity and low toxicity. Here, we examined the potency of Ps-K18, aiming to develop antibiotics derived from bioactive peptides for the treatment of Gram-negative sepsis. We first investigated the antibacterial mechanism of Ps-K18 based on confocal micrographs and field emission scanning electron microscopy, confirming that Ps-K18 targets the bacterial membrane. Anti-inflammatory mechanism of Ps-K18 was investigated by secreted alkaline phosphatase reporter gene assays and RT-PCR, which revealed that Ps-K18 activates innate defense via Toll-like receptor 4-mediated nuclear factor-kappa B signaling pathways. Moreover, we investigated the antiseptic effect of Ps-K18 using a lipopolysaccharide or *Escherichia coli* K1-induced septic shock mouse model. Ps-K18 significantly reduced bacterial growth and inflammatory responses in the septic shock model. Ps-K18 showed low renal and liver toxicity and attenuated lung damage effectively. This study suggests that Ps-K18 is a potent peptide antibiotic that could be applied therapeutically to Gram-negative sepsis.

## 1. Introduction

Naturally occurring bioactive peptides in various organisms are selective and effective cellular signaling molecules that play an important role either directly or indirectly in physiological processes. The peptides released through systems such as food processing or microbial fermentation play physicochemical roles to regulate important processes and exert beneficial effects on body functions [1]. Most peptides bind certain cell surface receptors such as G protein-coupled receptors (GPCRs) or ion channels, causing intracellular effects [2]. They can act as hormones, neurotransmitters, growth factors, ion channel ligands, or anti-infectious agents, and accordingly, these characteristics have become valuable to treat diseases that could not be cured previously [2,3]. Recently, bioactive peptides derived from nature have received attention due to their pharmacological effects and clinical potential [2,4]. For example, Lyxumia^®^ (Lixisenatide), an analogue of glucagon-like peptide 1 (GLP-1) isolated from Gila monster venom, was launched by Sanofi and is a GLP-1 receptor agonist for the treatment of type II diabetes that received approval from the FDA [5,6]. Further, Ile-Pro-Pro (IPP) and Val-Pro-Pro (VPP) are found in milk fermented with *Lactobacillus helveticus*, and are known as angiotensin-converting enzyme inhibitors that lower blood pressure [7]. Currently, many peptide drugs approved by the FDA have been supplied to the market and the number of peptide drugs under clinical development is steadily increasing. [2,6].

Various antibiotics have been identified since the discovery of penicillin, and proven effective in treating bacterial infections [8]. Imipenem, the first clinically available carbapenem antibiotic, and gentamicin, one of aminoglycoside antibiotics, are highly efficacious antibiotics against Gram-negative bacteria [9,10]. However, emergence of their multidrug-resistant (MDR) Gram-negative bacteria is a growing threat and the development of new antibiotic drugs are necessary [11,12]. Antimicrobial peptides (AMPs), molecules that have come under the spotlight as new antibiotics, are important components of the immune system found in all living organisms [13]. AMPs not only effectively inhibit bacterial infections but also exert an immunosuppressive effect and have potential as future therapeutics. However, these peptides have some limitations in that they are difficult to use directly as a treatment due to their poor physicochemical stability and cytotoxicity [2]. Polymyxin E, also known as colistin, shows potent antimicrobial activities against Gram-negative bacteria but it is used as a last resort due to kidney toxicity [4,14]. The decrease in toxicity mediated by chemical and sequence modification suggests an important strategy for the development of bioactive peptide-derived antibiotics [4]. Therefore, the design of highly functional antimicrobial peptides based on structure–activity relationships is expected to provide new possibilities for the development of antibiotics, since structural features such as hydrophobic and amphipathic properties affect their activities [15,16].

With the emergence of MDR Gram-negative bacteria and the failure of most new antibiotics in clinical trials, the development of new antibiotics has great importance in Gram-negative sepsis treatment [17]. Sepsis, a disease caused by bacterial infections, still has a high mortality rate of 35%–45% despite advances in antibiotic development and early targeted treatments [18,19]. Sepsis is caused mainly by the host’s reaction to lipopolysaccharide (LPS), an endotoxin [20]. LPS is located in the outer membrane of Gram-negative bacteria and is released in the infected host. This molecule is recognized by Toll-like receptors (TLRs) as a pathogen-associated molecular pattern (PAMP). TLR4 is activated by lipid A of LPS to induce the release of pro-inflammatory cytokines, which are important to activate the host immune response. An inflammatory reaction activated by the bacterial infection can lead to sepsis, a systemic inflammatory response syndrome [21,22,23].

Pseudin-2 (Ps; GLNALKKVFQGIHEAIKLINNHVQ) is a naturally occurring AMP derived from the skin of the paradoxical frog *Pseudis paradoxa*. Ps has a linear amphipathic α-helix structure from Leu^2^ to Glu^24^ residues and has shown efficacy against various bacteria, but it exerts severe cytotoxicity against mammalian cells [13]. In previous studies, we designed a Ps-K18 analogue by substituting Lys for Leu^18^ of Ps, making it less toxic, while maintaining the antibacterial activity of the parent peptide [24]. However, the detailed antibacterial and anti-inflammatory mechanism of Ps-K18 in relation to TLR4 signaling needs to be elucidated further.

The objective of the current study was to elucidate the antibacterial and anti-inflammatory mechanisms and evaluate the benefits of Ps-K18 as a bioactive peptide against sepsis induced by LPS or bacterial infection. With these aims, we examined the ability of Ps-K18 to inhibit bacterial growth and inflammation in vitro and in vivo. We elucidated the mechanism underlying the high antibacterial potency of Ps-K18, as well as its anti-inflammatory activity through TLR4 signaling. We also demonstrated the beneficial effects of Ps-K18 in vivo by monitoring the recovery of damaged organs in mouse models of endotoxemia or *E. coli* K1-induced septic shock. Our research results might provide insight into development of potent bioactive peptides for the treatment of sepsis caused by bacterial infection.

## 2. Results

### 2.1. Properties of Ps-K18

Most AMPs target bacteria by permeabilizing the cell membrane, resulting in its loss of function [25]. Structural features such as net charge, helicity, hydrophobicity, and amphipathicity are important factors affecting their antibacterial activity including their interactions with bacterial membranes [26].

**Figure 1:**
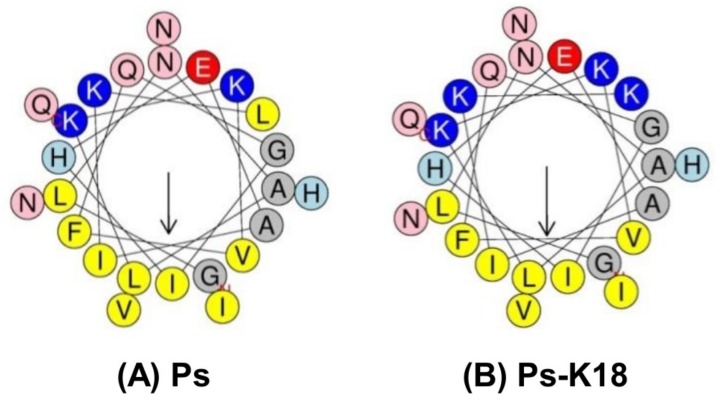
Helical-wheel diagrams of Ps and Ps-K18 using HeliQuest (http://heliquest.ipmc.cnrs.fr/). Helical-wheel diagrams of (**A**) Ps and (**B**) Ps-K18. Positively charged residues are shown in blue, negatively charged residues in red, and hydrophobic residues in yellow at the bottom of the wheel. In addition, Gly and Ala are shown in gray, Asn and Gln in pink, and His in sky blue. The arrows are presented based on the helical hydrophobic moment.

Thus, design based on the structure–activity of peptides is one of the most basic methods for developing new antibiotics [15,16]. Net charge is an important factor for the early interaction with negatively-charged bacterial membranes [27]. The solution structure of Ps has been determined as a linear α-helix from Leu^2^ to Glu^24^ residues and an amphipathic structure wherein one side is hydrophobic and the other side is hydrophilic with only a +2 net charge [24]. These specific structural features of Ps result in high cytotoxicity against mammalian cells, as well as bacteria. In our previous study, we designed Ps-K18, increasing the cationicity to +3 by replacing Leu^18^ between hydrophilic and hydrophobic sides with a Lys residue in the amphipathic α-helix (Table 1, Figure 1B); this resulted in lower toxicity but the maintenance of Ps antibacterial activity [24]. Peptide properties of Ps and Ps-K18 are listed in Table 1. The helical-wheel diagrams of Ps and Ps-K18 in Figure 1 show that replacing the Leu^18^ of Ps with Lys resulted in increased amphipathicity of the α-helical structure and cationicity, as well as decreased hydrophobicity. Decreased hydrophobicity can also reduce the antimicrobial activity of AMPs, whereas increased hydrophobicity can kill not only bacteria but also eukaryotic cells [27]. 

### 2.2. Antibacterial Activity

Previous studies showed high antibacterial activity of Ps and Ps-K18 against broad-spectrum Gram-negative bacteria [24]. We confirmed the antibacterial activities of the peptide only against Gram-negative bacteria and multidrug-resistant (MDR) Gram-negative bacteria to conduct further experiments on Ps-K18. Antibacterial activities were examined for four Gram-negative bacteria (*Escherichia coli* KCTC1682, *E. coli* K1, *Pseudomonas aeruginosa* KCCM11328, and *Acinetobacter baumannii* KCCM 40203) and three MDR Gram-negative bacteria (*E. coli* CCARM 1229, *P. aeruginosa* CCARM 2003, *A. baumannii* CCARM 12010). The antimicrobial activities of Ps and Ps-K18 are summarized in Table 2. Melittin, with high antibacterial activity and toxicity, was used as a control to compare antibacterial activity. Ps-K18 had similar antibacterial activity to Ps against standard and MDR Gram-negative bacteria. These results indicated that Ps-K18 is as effective as Ps against Gram-negative bacteria, and the results revealed that Ps-K18 is a potent antibiotic against MDR strains as well as standard strains.

### 2.3. Visualization of the Interaction Between E. coli and Ps Peptides based on Confocal Micrographs and Field Emission Scanning Electron Microscopic (FE-SEM) Micrographs

**Figure 2:**
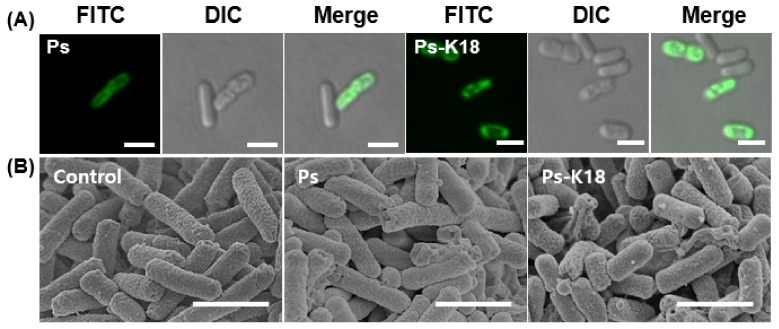
Confocal and scanning electron micrographs of *E. coli* treated with Ps and Ps-K18. (**A**) Confocal micrographs of Ps and Ps-K18 at a 1× MIC after 1 h incubation. The scale bar is 2 µm. (**B**) FE-SEM micrographs of control, no peptide treatment, and Ps and Ps-K18 (1× MIC) after 4 h incubation. The scale bar is 2 µm.

Ps and Ps-K18 showed high depolarization activity against *E. coli* as the concentration increased, implying that Ps and Ps-K18 target the bacterial membrane, in our previous study [24]. We further investigated the interaction between Ps peptides and bacteria. We first investigated the interaction between *E. coli* and FITC-labeled Ps or Ps-K18 by confocal microscopy (Figure 2A). For this, we treated *E. coli* with each peptide at the 1× MIC for 1 h. The Ps peptides were mainly localized on the surface of the *E. coli* membrane and no fluorescence was observed inside the cell.

The results suggested that Ps peptides attached to the surface of the bacterial membrane and caused disruption of the *E. coli* membrane. These results agree with the previous results obtained from dye leakage and depolarization experiments, indicating that Ps and Ps-K18 target the bacterial membrane [24]. We next visualized membrane damage induced by Ps or Ps-K18 using FE-SEM (Figure 2B). *E. coli* cells treated with a 1× MIC of Ps for 4 h were wrinkled and contracted, whereas control cells had a normal oval shape (Figure 2B). *E. coli* cells treated with a 1× MIC of Ps-K18 were crumpled and contracted, similar to that with Ps treatment. This proved that Ps-K18, which retained high antibacterial activity similar to that of Ps, effectively interacts with the membrane to cause fatal damage to the bacterium. 

### 2.4. Cytotoxicity of Ps and Ps-K18 In Vitro and In Vivo 

**Figure 3:**
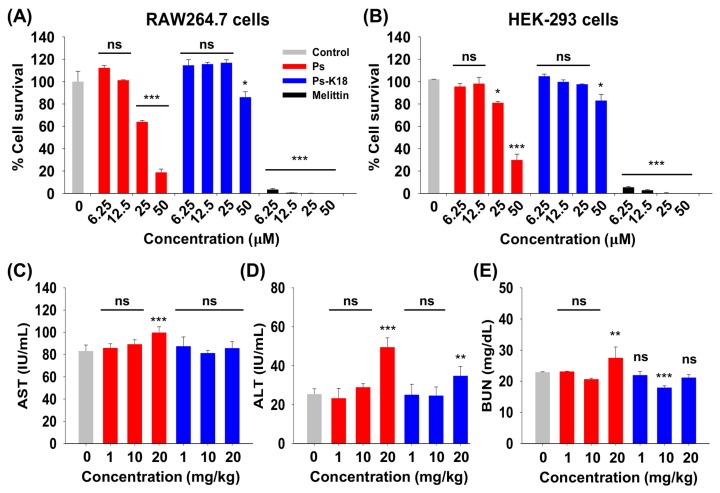
Cytotoxicity of Ps and Ps-K18 in vitro and in vivo. Concentration-dependent toxicities of pseudin-2 (Ps) and Ps-K18 against (**A**) RAW264.7 cells and (**B**) HEK-293 cells. (**C**) Aminotransferase (AST); (**D**) alanine aminotransferase (ALT); and (**E**) blood urea nitrogen (BUN) levels in mouse serum. The error bars represent means ± standard error of the mean. (*p < 0.05; **p < 0.01; ***p < 0.001; ns. represents no significance).

Since toxicity of highly active peptides has always been a limit for the therapeutic application of peptide drugs, we examined the toxicity of Ps and Ps-K18 against mammalian cells [2]. The survival rates of mouse macrophage RAW264.7 cells and human embryonic kidney HEK-293 cells are shown in Figure 3A,B. Melittin showed extremely high cytotoxicity at lower concentrations. At 50 µM, survival rates were 18% and 31% for Ps and 82% and 84% for Ps-K18 against RAW264.7 cells and HEK-293 cells, respectively. 

We then evaluated the in vivo cytotoxicity of Ps peptides by measuring levels of alanine aminotransferase (ALT), aspartate aminotransferase (AST), and blood urea nitrogen (BUN) in the serum of mice. ALT and AST are used as indicators of liver damage, whereas BUN is used to assess kidney damage [28]. When Ps was injected into mice, levels of AST, ALT, and BUN increased with higher concentrations (Figure 3C–E). However, Ps-K18 did not increase the level of AST and BUN as concentrations increased, but increased ALT only at very high dose, 20 mg/kg, although much lower than Ps. These results support that Ps-K18 has improved potential as a therapeutic agent because it is less toxic than the Ps while maintaining high antibacterial activity.

### 2.5. Specificity of Ps-K18 towards Various TLRs

TLRs, which play an important role in the innate immune system, are activated by the specific agonist molecules, causing an inflammatory reaction [29]. Pam_2_CSK_4_ activates TLR2/6 signaling and Pam_3_CSK_4_ activates the TLR2/1 inflammation response. Polyinosinic:polycytidylic acid (Poly(I:C)) activates TLR3, and LPS activates TLR4 inflammatory signaling. TLR7 and TLR9 can be activated by imiquimod and ODN1826, respectively [30].

**Figure 4:**
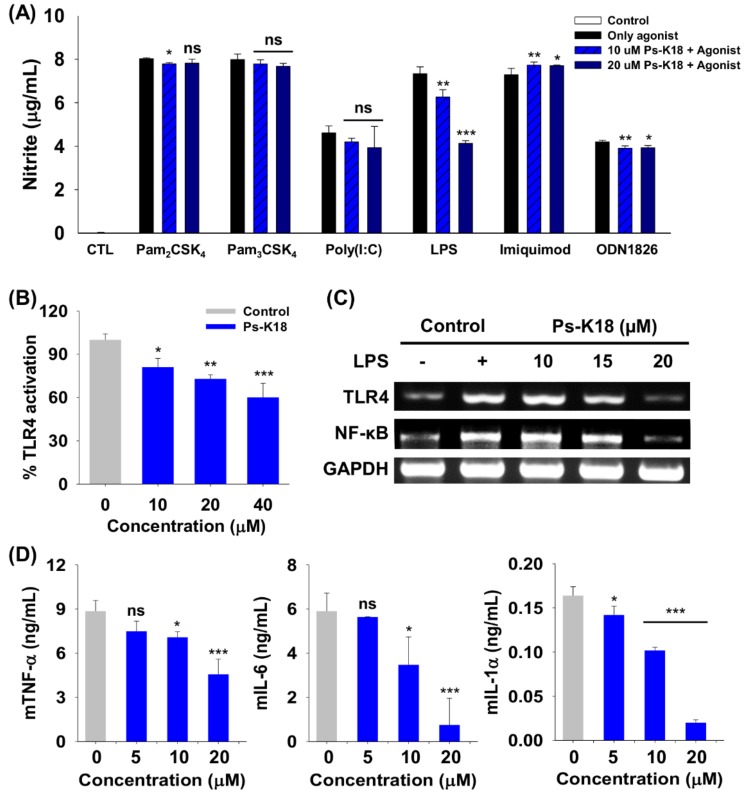
Specificity of Ps-K18 toward various Toll-like receptors (TLRs) and TLR-specific agonists, and anti-inflammatory activity of Ps-K18. (**A**) Specificity of Ps-K18 for TLRs and TLR-specific agonists that selectively activate TLRs, as determined by measuring nitrite production in RAW264.7 cells. Different TLRs were selectively activated by Pam_2_CSK_4_ (200 ng/mL), Pam_3_CSK_4_ (200 ng/mL), Poly(I:C) (2 µg/mL), LPS (20 ng/mL), imiquimod (1 µg/mL), and ODN1826 (20 µg/mL). Control is the data without agonist treatment. (**B**) Dose-dependent reduction in secreted alkaline phosphatase reporter gene (SEAP) activity by Ps-K18 in LPS (20 ng/mL)-stimulated HEK-Blue™ hTLR4 cells. (**C**) Inhibitory effect of Ps-K18 on inflammation-related gene expression in LPS (50 ng/mL)-stimulated RAW264.7 cells. The cells were pre-treated with Ps analogues for 1 h and then treated with LPS for 12 h. (**D**) Inhibitory effects on the production of inflammatory cytokines tumor necrosis factor-α (TNF-α), interleukin (IL)-6, and IL-1α by Ps-K18 (5 µM, 10 µM, and 20 µM) in LPS (20 ng/mL)-stimulated RAW264.7 cells. The error bars represent means ± standard error of the mean (*p < 0.05; **p < 0.01; ***p < 0.001; ns. represents no significance).

We investigated the specificity of Ps-K18 towards various TLRs. For this, we treated RAW264.7 cells with Ps-K18 and known TLR-specific agonists (Figure 4A). Ps-K18 significantly suppressed TLR4-mediated signaling in RAW264.7 cells by 15% and 43% based on LPS-induced nitrite production at 10 µM and 20 µM, respectively. Ps-K18 did not effectively inhibit nitrite production in the presence of other agonists (Pam_2_CSK_4_, Pam_3_CSK_4_, polyinosinic:polycytidylic acid (Poly(I:C)), imiquimod, ODN1826) and TLRs, implying that Ps-K18 selectively modulates LPS-induced TLR4 signaling.

### 2.6. Ps-K18 Suppresses Inflammation Response through TLR4-Mediated Signaling in RAW264.7 Cells and HEK-Blue^TM^ hTLR4 Cells

To investigate the mechanism underlying the anti-inflammatory activity of Ps-K18, we performed a secreted alkaline phosphatase reporter (SEAP) gene assay (Figure 4B). LPS, released from Gram-negative bacteria, stimulates cells by activating TLR4-mediated signaling to induce inflammatory cytokine and nitric oxide release, leading to sepsis [31]. HEK-Blue^TM^ hTLR4 cells that express TLR4 contain an SEAP gene located downstream from the nuclear factor-kappa B (NF-κB) promoter, which results in TLR4-mediated reporter activation when the cells are stimulated with LPS [32]. Ps-K18 inhibited 19%, 27%, and 40% of SEAP activity efficiently at 10, 20, and 40 µM, respectively. 

TLR4 activated by LPS induces NF-κB phosphorylation. Further, phosphorylated NF-κB is translocated to the nucleus and activates inflammatory cytokine gene transcription [30]. We also confirmed that the gene expression of TLR4 and NF-κB can be inhibited by Ps-K18 in LPS-stimulated RAW264.7 cells through Reverse transcription polymerase chain reaction (RT-PCR). By increasing the concentration of Ps-K18, it effectively suppressed the gene expression of TLR4 and NF-κB (Figure 4C). When we quantified relative gene expression of TLR4 and NF-κB using ImageJ software, they were decreased by approximately 83% and 85%, respectively, at 20 µM. These results showed that Ps-K18 exerts anti-inflammatory activity by inhibiting LPS-induced TLR4-mediated signaling. 

We further investigated the anti-inflammatory activity of Ps-K18 to confirm inhibitory effects on TLR4-mediated signaling (Figure 4D). Ps-K18 effectively inhibited the production of the inflammatory cytokines mTNF-α, mIL-6 and mIL-1α depending on concentration. Ps-K18 suppressed cytokines mTNF-α by 49%, mIL-6 by 87% and mIL-1α by 88%, at 20 µM. These results showed that Ps-K18 has potentially high anti-inflammatory activity that can effectively inhibit inflammatory responses with low cytotoxicity. 

### 2.7. Antisepsis Effect of Ps-K18 in LPS-Induced Endotoxemia Mouse Model

**Figure 5:**
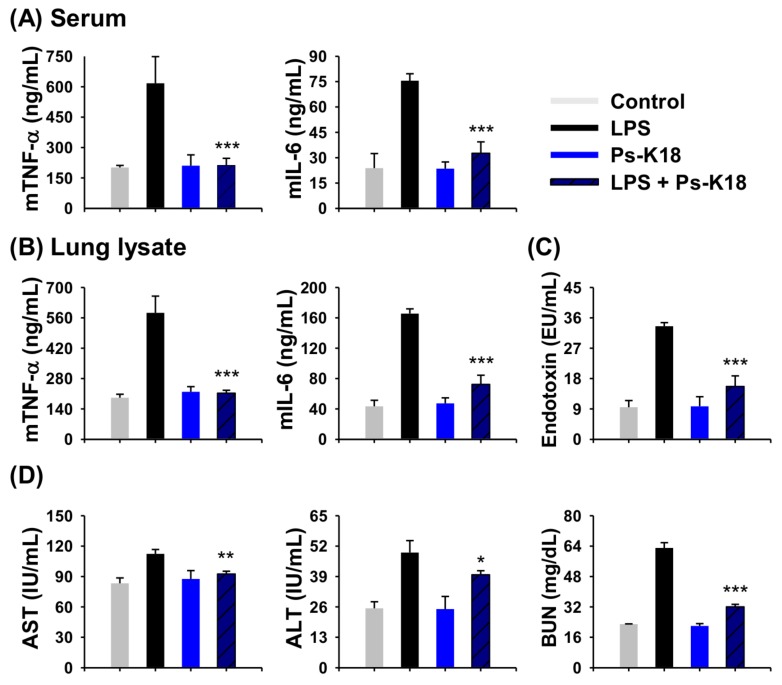
Effects of Ps-K18 on LPS-induced endotoxemia mouse model. (**A**) Inhibition of cytokine production (mTNF-α and mIL-6) in the serum. (**B**) Inhibition of cytokine production (mTNF-α and mIL-6) in lung lysates. (**C**) Reduction of LPS endotoxin by Ps-K18 in serum of the LPS-induced sepsis model. (**D**) AST, ALT, and BUN levels in the mouse septic shock model induced by LPS. BALB/c mice were pre-treated with 1 mg/kg of peptide, followed by intraperitoneal injection with 15 mg/kg of LPS. Data are presented as means ± standard errors of the mean (*p < 0.05; **p < 0.01; ***p < 0.001; ns. represents no significance) compared to LPS-injected group. All measurements were obtained five times.

Since Ps-K18 is a potent peptide with high antibacterial and anti-inflammatory activity in vitro, we next confirmed its potential as a therapeutic agent to treat sepsis in vivo. For this, we first investigated the anti-inflammatory activity of Ps-K18 using an endotoxemia mouse model induced by LPS. LPS is an endotoxin located in the outer membrane of Gram-negative bacteria, and triggers a severe inflammatory response, causing sepsis in the body [20]. Cytokine storms that can lead to organ damage and death aggravate the inflammatory response during endotoxemia [33]. The group injected only with LPS showed high levels of inflammatory cytokine expression (Figure 5A,B). Only the Ps-K18 treatment group showed similar results to the control group (Figure 5). Ps-K18 resulted in a marked reduction in inflammatory cytokines mTNF-α and mIL-6. In the serum, Ps-K18 effectively reduced inflammatory cytokines mTNF-α by 65% and mIL-6 by 57% when Ps-K18 was injected with LPS in mice. (Figure 5A). Ps-K18 also inhibited inflammatory cytokines mTNF-α by 64% and mIL-6 by 56% in the lung (Figure 5B). 

We also tested whether Ps-K18 can remove LPS endotoxin in vivo by performing a limulus amebocyte lysate (LAL) assay that measures the amount of endotoxin in the sample (Figure 5C). Ps-K18 reduced the endotoxin levels by 53% when injected with LPS. These results revealed that Ps-K18 can effectively decrease endotoxin levels in vivo. In addition, the levels of AST, ALT, and BUN were increased by LPS but were effectively reduced by Ps-K18 treatment (Figure 5D). Levels of AST, ALT, and BUN were reduced by 18%, 19%, and 49%, respectively, when Ps-K18 was injected with LPS into the mice. These results showed that Ps-K18 treatment can improve the functions of organs damaged by the endotoxin LPS in vivo.

### 2.8. Antisepsis Effect of Ps-K18 Based on an E. coli K1-Induced Mouse Model of Septic Shock 

**Figure 6:**
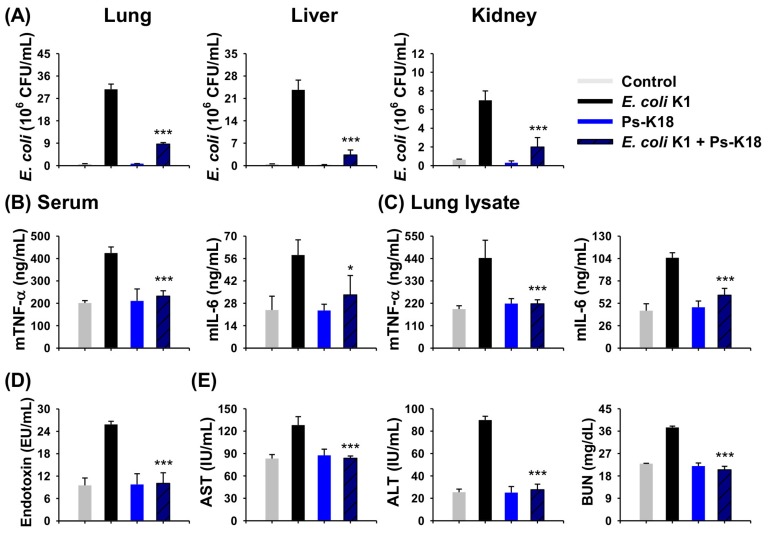
Effects of Ps-K18 on septic shock in mice induced by *E. coli* K1. (**A**) Inhibition of bacterial growth in the lung, liver, and kidney of the septic shock model. BALB/c mice were pre-treated with 1 mg/kg of peptide, followed by intraperitoneal injection with *E. coli* K1 (1 × 10^6^ CFU/mouse). (**B**) Inhibition of cytokine production (mTNF-α and mIL-6) in the serum. (**C**) Inhibition of cytokine production (mTNF-α and mIL-6) in lung lysates. (**D**) Reduction of LPS endotoxin by Ps-K18 in serum of the *E. coli* K1-induced sepsis model. (**E**) AST, ALT, and BUN levels in the mouse septic shock model induced by *E. coli* K1. Data are presented as means ± standard errors of the mean (*p < 0.05; **p < 0.01; ***p < 0.001; ns. represents no significance) compared to *E. coli* K1-injected group. All measurements were obtained five times.

Since sepsis is one of diseases caused by bacterial infection and there are urgent needs to develop new antiseptic agents [18,19], we examined the potency of Ps-K18 as a candidate antiseptic peptide drug. We used an intact *E. coli* K1-induced septic shock mouse model to confirm its potential as a therapeutic agent for sepsis. Ps-K18 reduced the number of bacteria in the mouse lung, liver, and kidney by approximately 73%, 87%, and 71%, respectively. This indicated that Ps-K18 can effectively inhibit the growth of bacteria, *E. coli* K1 in vitro and in vivo (Figure 6A). Because Ps-K18 had high anti-inflammatory activity in vitro, we then measured the in vivo anti-inflammatory activity using serum and lung lysates of mice with *E. coli* K1-induced septic shock. We detected high levels of mTNF-α and mIL-6 in the experimental group only treated *E. coli* K1. However, Ps-K18 reduced TNF-α levels by approximately 45% and mIL-6 by 43% in the serum when injected with *E. coli* K1 (Figure 6B). Ps-K18 also reduced mTNF-α levels by 51% and mIL-6 by 41% in the lungs of septic mice (Figure 6C). 

We also performed LAL assays to confirm the potency of Ps-K18 using the *E. coli* K1-induced sepsis model. Ps-K18 reduced levels of endotoxin by 61% when administered with *E. coli* K1 (Figure 6D). The levels of AST, ALT, and BUN were also measured using the blood of mice with sepsis induced by *E. coli* K1. Ps-K18 reduced the levels of AST by 35%, ALT by 69%, and BUN by 45%, when injected with *E. coli* K1 (Figure 6E). This showed that Ps-K18 is effective for the treatment sepsis by inhibiting the growth of bacteria and restoring liver and kidney function after damage mediated by bacterial infection in vivo.

### 2.9. Ps-K18 Treatment Effectively Suppresses Polymorphonuclear Lymphocyte Infiltration in LPS-Induced Endotoxemia Mouse Model and E. coli K1-Induced Mouse Model

**Figure 7:**
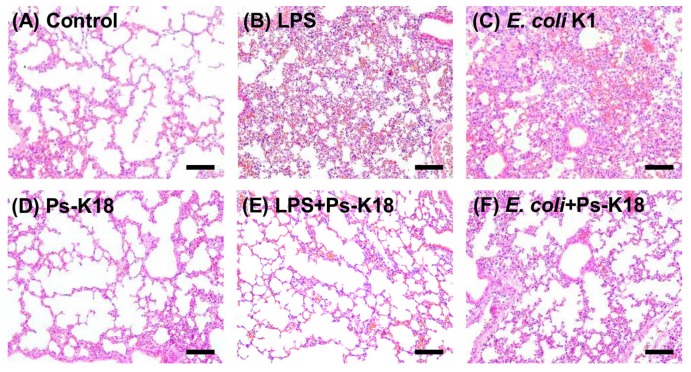
Effect of Ps-K18 on neutrophil infiltration in the lungs of septic shock mouse induced by LPS or *E. coli* K1. Lung morphology of (**A**) control, no peptide treatment, (**B**) LPS (15 mg/kg), (**C**) *E. coli* K1 (1 × 10^6^ CFU/mouse), (**D**) Ps-K18 (1 mg/kg), (**E**) LPS + Ps-K18, injected with both LPS and Ps-K18, and (**F**) *E. coli* K1 + Ps-K18, injected with both *E. coli* K1 and Ps-K18. LPS or *E. coli* K1 was pre-injected in mice for 1h, and then Ps-K18 injected in mice. The scale bar is 100 µm.

We also pathologically investigated the lung tissues obtained from LPS-induced endotoxemia and *E. coli* K1-induced mouse models. Capillary-alveolar barrier dysfunction occurs during the infiltration of polymorphonuclear lymphocytes (PMNs) into the lungs, which is followed by pulmonary edema with impaired pulmonary gas exchange [34,35]. PMNs are associated with lung damage and are an important cause of acute lung injury, which results in high mortality and morbidity during sepsis. Therefore, reducing the number of PMNs is of great importance for the treatment of sepsis [36]. Figure 7 shows the pathological characteristics of lung damage induced by LPS or *E. coli* K1 infection, which were improved by Ps-K18. Control mice, not treated with LPS or *E. coli* K1, and Ps-K18 mice, only injected with Ps-K18, showed normal lung morphology, whereas LPS or *E. coli* K1 treatment induced morphological changes and PMN infiltration (Figure 7A–D). However, Ps-K18 treatment effectively suppressed PMN infiltration and significantly improved lung morphology, similar to that observed in the control group (Figure 7E,F). All results obtained from the septic shock model revealed that Ps-K18 can be used to treat sepsis in vivo.

## 3. Discussion

Sepsis is a highly lethal disease that induces severe inflammatory reactions throughout the body [37,38]. In particular, sepsis caused by MDR Gram-negative bacteria is more difficult to treat as it is associated with reductions in the efficacy of existing antibiotics [39]. To meet these social needs, new drug development is urgently needed. Among the antibiotics that have been developed, peptide antibiotics have received attention as new therapeutic agents due to their outstanding potency with strong antibacterial activity against drug-resistant strains and immunosuppressive properties, and act by controlling host defense systems [4,15,24,40]. Despite these properties, low cell selectivity and high cytotoxicity of AMPs are obstacles that impede their use in disease treatment. Because the activity of AMPs is affected by physiochemical properties such as net charge helicity, hydrophobicity, and amphipathicity, the optimization of these properties is one of the most common means to develop highly-functional AMPs [41,42]. Colistin, also called polymyxin E, is a peptide antibiotic that represents the last resort against MDR Gram-negative sepsis due to its high toxicity [43,44]. Therefore, there are rapid needs for the development of new peptide antibiotics that can protect against bacterial infection with high safety; this offers the potential for new solutions to eliminate rapidly emerging multidrug-resistant bacterial strains. 

AMPs have a variety of net charge ranges from +2 to +9 [45]. The cationicity of peptides is known to be essential for their insertion into and destruction of cell membranes because it leads to structural instability in bacteria due to interactions with their negatively charged membrane, thereby improving the antibacterial activity [42,46,47]. It was reported that a truncated Ps analogue, with increased net charge, has excellent inhibitory effects on bacterial growth [48]. In a previous study, we designed Ps-K18 (+3) with a Lys substitution for Leu^18^ in Ps and showed that it has high antibacterial activity and low toxicity [24]. Here, we investigated the detailed antibacterial mechanism of Ps-K18 as a potent peptide antibiotic, using confocal micrographs and SEM analysis (Figure 2). Ps peptides were located at the surface of the membranes of *E. coli* (Figure 2A) and the morphology of *E. coli* changed into the wrinkled and disrupted shape after Ps or Ps-K18 treatment (Figure 2B). These results revealed the antibacterial mechanism of Ps or Ps-K18, which effectively inhibited the growth of bacteria by targeting bacterial membranes, confirming our previous results obtained from dye leakage and depolarization assays against various membranes [24].

We checked the toxicity of Ps peptides for its safe therapeutic application. Ps-K18 had very low toxicity compared to Ps in vivo and in vitro (Figure 3). LPS released from Gram-negative bacteria activates TLR4 to induce the release of pro-inflammatory cytokines, which are important to activate the host immune response [20]. Because serious inflammatory responses can induce sepsis, there is extensive interest in peptide antibiotics that suppress inflammatory responses and inhibit bacterial growth. Furthermore, Ps-K18 exerted significant effects in specifically suppressing LPS-induced TLR4 signaling, among various TLRs (Figure 4A). We then investigated the anti-inflammatory activity of Ps-K18 in LPS-stimulated RAW264.7 cells (Figure 4B–D). Ps-K18 effectively suppressed the production of inflammatory TNF-α, IL-6, and IL-α (Figure 4D). Our previous study showed that Ps and Ps-K18 bind TLR4 tightly with 0.8 and 1.5 μM affinity, respectively [24]. The detailed mechanism underlying the anti-inflammatory activity of Ps-K18 was demonstrated by SEAP assays and RT-PCR, implying that Ps-K18 has remarkable anti-inflammatory activity by suppressing TLR4-mediated NF-κB signaling pathways.

We demonstrated that Ps-K18 is a potent peptide antibiotic in vitro, with antibacterial activity that effectively relies on interactions with bacterial membranes to induce the death of bacteria, as well as anti-inflammatory effects that inhibit TLR4-mediated NF-κB signaling pathways. To prove that Ps-K18 is an effective antibiotic not only in vitro but also in vivo, we conducted in vivo experiments using LPS-induced endotoxemia or *E. coli* K1-induced septic shock mouse model. Sepsis, mainly induced by LPS released from bacteria, activates all components of the immune system, including various blood cells, endothelial cells, and bone marrow, to remove infecting bacteria from the body. During this process, major organs such as the lungs, abdomen, and urinary tract appear to be damaged by the release of different mediators [49]. The in vivo efficacy of Ps-K18 was examined by monitoring organ injury in LPS-induced endotoxemia and *E. coli* K1-induced septic shock models (Figure 5 and Figure 6). Importantly, Ps-K18 effectively inhibited the growth of Gram-negative bacteria and the associated production of inflammatory cytokines mTNF-α and mIL-6, ameliorated liver and kidney damage induced by infection, and reduced the amount of endotoxin LPS in serum. From LAL assay in septic shock model, we showed that Ps-K18 can effectively decrease endotoxin LPS levels in vivo. Antimicrobial peptides such as polymyxin B and LL-37 are well known LPS neutralizing agents which can prevent the initiation of the inflammatory responses [50]. LPS in the blood can stimulate the expression of inflammatory cytokines, resulting in septic shock [51]. In case of Gram-negative infection, where the innate immunity mediator TLR4 is already significantly enhanced, antiseptic agent should be able to suppress the LPS or bacteria-induced TLR4 activation which eventually causes septic shock. As shown in this study, Ps-K18 can be a potent antiseptic agent, having both LPS neutralizing ability as well as inhibiting ability of systemic TLR4-mediated inflammatory signaling by blocking TLR4/NF-κB activation in septic shock mouse model. Our results confirmed that Ps-K18 could be an excellent therapeutic agent for treatment of sepsis.

## 4. Materials and Methods

### 4.1. Peptide Synthesis

All peptides listed in Table 1, synthesized by solid-phase synthesis using N-(9-fluorenyl) methoxycarbonyl solid-phase synthesis technique, were purified by reversed-phase preparative high-performance liquid chromatography [52]. Purities of the peptides (>95%) were measured using an analytical C_18_ column. Molecular masses and retention times of peptides were determined by matrix-assisted laser-desorption ionization-time-of-flight (MALDI-TOF) mass spectrometry at Korea Basic Science Institute (KBSI, Ochang, Korea).

### 4.2. Antibacterial Activity

We purchased *E. coli* KCTC 1682, *P. aeruginosa* KCCM 11328, *A. baumannii* KCCM 40203 from the Korean Collection for Type Cultures (KCTC) (Taejon, Korea), and Korean Culture Center of Microorganisms (KCCM) (Seoul, Korea). MDR Gram-negative bacteria (*E. coli* CCARM 1229, *P. aeruginosa* CCARM 2003, *A. baumannii* CCARM 12010) were obtained from the Culture Collection of Antibiotic-Resistant Microbes (CCARM) at Seoul Women’s University in Korea. We were kindly provided *E. coli* K1 strain RS218 (O18:K1:H7) by Jang-Won Yoon of Kangwon National University (Gangwon-do, Korea). MIC of peptides was determined by the broth microdilution assay using 1% peptone media, as reported previously [24]. MIC was determined by the average value of three independent experiments. Peptides were diluted using 1% peptone media treated with 2 × 10^5^ CFU/mL of bacterial suspensions in 1% peptone media for 16 h at 37 °C.

### 4.3. Confocal Microscope Analysis

When *E. coli* KCTC 1682 became a mid-log phage, it was washed and diluted in 10 mM PBS buffer. Bacterial suspensions (1 × 10^7^ CFU/mL) were incubated with 1× MIC of peptides for 1 h. After 1 h, the cells were washed three times using 10 mM PBS buffer and visualized with a confocal laser scanning microscope (LSM 800; Carl Zeiss, Oberkochen, Germany).

### 4.4. FE-SEM Analysis

We visualized the membrane damage of *E. coli* KCTC 1682 by FE-SEM to confirm the antibacterial mechanisms. The method was the same as a previously reported method [28]. The 1 × MIC of peptide treated to *E. coli* KCTC 1682 was diluted in 10 mM PBS buffer to an OD_600_ of 0.2 and incubated for 4 h at 37 °C. After the fixation and dehydration step, the cells were visualized with a FE-SEM (SU8020; Hitachi, Tokyo, Japan).

### 4.5. Cytotoxicity In Vitro

RAW264.7 cells and HEK-293 cells were purchased from Korean cell line bank (Seoul, Korea). The cytotoxicity of peptides towards RAW264.7 cells and HEK-293 cells were evaluated by Cell Counting Kit-8 purchased from Abbkine Scientific (Wuhan, China). The assay was the same as previously reported method [28,53].

### 4.6. Specificity Against TLRs Selectively Activated by Agonists

The specificity of peptides was assessed by measuring nitrate production in RAW264.7 cells after activating different TLRs. Agonists including Pam_2_CSK_4_ (200 ng/mL), Pam_3_CSK_4_ (200 ng/mL), Poly(I:C) (2 µg/mL), LPS (20 ng/mL), and ODN1826 (20 µg/mL) were purchased from Invivogen (San Diego, CA, USA). Imiquimod (1 µg/mL) was purchased from Sigma-Aldrich (St. Louis, MO, USA). The method was similar to that previously reported [30]. We pretreated cells with Ps peptide for 1 h. Next, we added the aforementioned agonists at the indicated concentrations. To confirm the specificity of peptides, nitrate production was detected at an absorbance of 540 nm, as previously reported [28].

### 4.7. Quantification of Inflammatory Cytokine Production in LPS-Stimulated RAW264.7 Cells

ELISA assay was used to check the level of inflammatory cytokine. Raw264.7 cells plated at a density of 1 × 10^5^ cells/well were stimulated with 20 ng/mL LPS of *E. coli* O111:B4 (Sigma-Aldrich, St. Louis, MO, USA) with or without peptide for 16 h. The inhibition effect of peptide on mTNF-α, mIL-6 and mIL-1α expression, which belong to inflammatory cytokines, was measured by enzyme-linked immunosorbent assays (ELISA; R&D Systems, Minneapolis, MN, USA), as previously reported [54]. Glyceraldehyde 3-phosphate dehydrogenase (GAPDH) was used control in the RT-PCR. Relative intensity of the DNA bands was quantified by ImageJ software (Version 1.52q, National Institutes of Health, Bethesda, MD, USA).

### 4.8. Inhibition Effect of Ps-K18 on TLR4-Mediated Inflammatory Response in LPS-Stimulated HEK-Blue^TM^ hTLR4 and RAW264.7 Cells

HEK-Blue^TM^ hTLR4 cells (InvivoGen, San Diego, CA, USA) were plated at a density of 2.5 × 10^4^ cells/well with Ps-K18 in HEK-Blue detection media (InvivoGen, San Diego, CA, USA). After 1 h, the cells were stimulated by 20 ng/mL LPS and incubated for 8 h at 37 °C in a humidified 5% CO_2_ atmosphere. SEAP activity was determined by measuring absorbance at 630 nm.

RT-PCR was performed to measure inhibition activity of Ps-K18 on expression of TLR4 mediating inflammatory response signaling as previously reported [55]. The results visualized by UV illumination using Alpha Innotech gel documentation system (Alphaimager ^®^ HP; Alpha Innotech Corporation, San Leandro, CA, USA).

### 4.9. Sepsis Mouse Model

Female BALB/c mice (6-week-old) were purchased from Orient (Daejeon, Korea). All mice were housed under specific pathogen-free (SPF) conditions and allowed free access to food and water in a temperature- and humidity-controlled environment. All mice were used for LPS or *E. coli* K1-induced sepsis model. *E. coli* K1 possesses K1 capsular polysaccharide antigen which is an essential virulence determinant that protects the bacteria from immune attack [56,57]. All procedures were approved by the Institutional Animal Care and Use Committee (IACUC) of Konkuk University, South Korea (IACUC number: KU18163-1, approved on 17 April 2019). 

### 4.10. Cytotoxicity In Vivo

Cytotoxicity was determined by detecting AST, ALT, and BUN levels in mice serum. They were detected five times independently using a standard kit available for purchase from Asan pharmaceutical (Seoul, Korea), as described previously [58]. The control group was only injected with PBS and the Ps or Ps-K18 groups were only administered peptide (1, 10, 20 mg/kg). After 16 h, the mice were sacrificed for analysis.

### 4.11. Measurement of Antiseptic Activity of Peptides in LPS-Induced Endotoxemia and E. coli K1 Septic Shock Mouse Model

To determine the antiseptic activity of Ps-K18, experiments were performed based on four different groups. The control group was only injected with PBS and the peptide group was only administered Ps-K18 (1 mg/kg). The LPS group was only injected with LPS (15 mg/kg) and the LPS + Ps-K18 group received an injection of LPS (15 mg/kg) 1 h after the administration of Ps-K18 (1 mg/kg). The *E. coli* group was only injected with *E. coli* K1 (1 × 10^6^ CFU/mouse), whereas the *E. coli* + Ps-K18 group received an injection of *E. coli* K1 (1 × 10^6^ CFU/mouse) 1 h after the administration of Ps-K18 (1 mg/kg). Mice were inoculated via intraperitoneal (i.p.) injection. After 16 h, the mice were sacrificed for analysis.

To measure cytokine levels in the serum or lung lysates of the sepsis model, we used ELISA kits (R&D Systems, Minneapolis, MN, USA), as reported previously [54]. The experiments were performed five times independently. We additionally confirmed the inhibitory effect of the peptide on endotoxin LPS using a LAL kit purchased from Lonza Group Ltd. (Allendale, NJ, USA), as previously reported [28]. We counted the number of bacteria in organ tissues, as previously reported, to determine whether Ps-K18 could inhibit the growth of bacteria in vivo [28]. Serum AST, ALT, and BUN levels were detected five times independently using a standard kit to confirm recovery ability of peptide for damaged organ by LPS or *E. coli* K1, as described previously [58]. To determine the suppressive effect of the peptide on neutrophil infiltration in vivo, tissue slides were produced, as previously described [59]. Briefly, lung tissues extracted from mice were fixed in 4% paraformaldehyde solution and dehydrated to produce paraffin blocks. The paraffin blocks were cut to an appropriate thickness to prepare tissue slides and the remaining paraffin was removed with xylene. The slides were then stained with hematoxylin and eosin and visualized with a microscope (Eclipse Ni; Nikon, Tokyo, Japan).

### 4.12. Statistical Analysis

All measurements were obtained at least three times and statistical analysis was calculated using Graphpad Prism (GraphPad Software Inc., La Jolla, CA, USA). One-way ANOVA followed by post-hoc Bonferroni tests (Prism 8.0) were used to compare with the experimental groups.

The error bars reveal standard error of measurement (±SEM). Values indicate statistically significance at *p < 0.05; **p < 0.01; ***p < 0.001; n.s. represents no significance.

## 5. Conclusions

Our study showed that Ps-K18 has low toxicity upon Lys substitution at Leu^18^ in Ps and functions through antibacterial mechanisms to restrict bacterial growth by targeting bacterial membranes and anti-inflammatory mechanism-inhibiting inflammatory reactions by suppressing LPS-induced TLR4 signaling with high specificity in vivo and in vitro. These properties led to excellent therapeutic effects in treating sepsis caused by endotoxin or *E. coli* K1. In our future studies, we must overcome obstacles such as the stability of the peptide in vivo, reduced activity mediated by proteases, cost, and the scaling of production of this peptide [60]. Developing methods to overcome these limitations will enable the application of Ps-K18 as a peptide drug derived from bioactive peptides. All results in this study clearly showed that Ps-K18 is a potential antimicrobial candidate that functions through dual mechanisms to suppress bacterial growth and inhibit inflammatory reactions in vitro and in vivo. Accordingly, Ps-K18 could be a potent peptide antibiotic for the effective treatment of sepsis caused by Gram-negative bacterial infections. This study may provide insight into design and development of a potent peptide drug which could be used as an effective treatment for sepsis and into a pharmaceutical application of peptide antibiotics.

## Figures and Tables

**Table 1:** Amino acid sequences and peptide properties.

Peptide	Sequence	Molecular Weight^a^	Net Charge	Hydrophobicity^b^ <H>
Ps	GLNALKKVFQGIHEAIKLINNHVQ	2685	+2	0.407
Ps-K18	GLNALKKVFQGIHEAIKKINNHVQ	2702	+3	0.295

^a^ The molecular weight (MW) was measured by mass spectroscopy. ^b^ Hydrophobicity <H> was calculated online at: http://heliquest.ipmc.cnrs.fr/cgi-bin/ComputParams.py.

**Table 2:** Antimicrobial activities of the Ps and Ps-K18 against standard bacterial strains and MDR bacterial strains.

MIC (µM)	Ps	Ps-K18	Melittin
Standard Gram-negative bacteria			
*E. coli* KCTC1682	4	4	4
*E. coli* K1	2	2	2
*P. aeruginosa* KCCM11328	4	4	8
*A. baumannii* KCCM40203	2	2	2
MDR Gram-negative bacteria			
*E. coli* CCARM 1229	2	2	1
*P. aeruginosa* CCARM 2003	2	2	2
*A. baumannii* CCARM 12010	2	2	1
GM^a^	2.57	2.57	2.86

^a^ The geometric means (GM) are the average values of minimum inhibitory concentrations (MICs).

## References

[1] Chakrabarti S., Guha S., Majumder K. (2018). Food-derived bioactive peptides in human health: Challenges and opportunities. Nutrients.

[2] Fosgerau K., Hoffmann T. (2015). Peptide therapeutics: Current status and future directions. Drug Discov. Today.

[3] Hayashi M.A., Ducancel F., Konno K. (2012). Natural peptides with potential applications in drug development, diagnosis, and/or biotechnology. Int. J. Pept..

[4] Hancock R.E., Chapple D.S. (1999). Peptide antibiotics. Antimicrob. Agents Chemother..

[5] Koppkubel S. (1995). International nonproprietary names (inn) for pharmaceutical substances. Bull. World Health Organ..

[6] Kaspar A.A., Reichert J.M. (2013). Future directions for peptide therapeutics development. Drug Discov. Today.

[7] Iwaniak A., Darewicz M., Minkiewicz P. (2018). Peptides derived from foods as supportive diet components in the prevention of metabolic syndrome. Compr. Rev. Food Sci. Food Saf..

[8] Williams K.J. (2009). The introduction of ’chemotherapy’ using arsphenamine-the first magic bullet. J. R. Soc. Med..

[9] Drusano G.L. (1986). An overview of the pharmacology of imipenem/cilastatin. J. Antimicrob. Chemother..

[10] Lin L., Wagner M.C., Cocklin R., Kuzma A., Harrington M., Molitoris B.A., Goebl M.G. (2011). The antibiotic gentamicin inhibits specific protein trafficking functions of the arf1/2 family of gtpases. Antimicrob. Agents Chemother..

[11] Codjoe F.S., Donkor E.S. (2017). Carbapenem resistance: A review. Med. Sci. (Basel).

[12] Moellering R.C., Wennersten C., Kunz L.J. (1976). Emergence of gentamicin-resistant bacteria: Experience with tobramycin therapy of infections due to gentamicin-resistant organisms. J. Infect. Dis..

[13] Reddy K.V., Yedery R.D., Aranha C. (2004). Antimicrobial peptides: Premises and promises. Int. J. Antimicrob. Agents.

[14] Falagas M.E., Grammatikos A.P., Michalopoulos A. (2008). Potential of old-generation antibiotics to address current need for new antibiotics. Expert Rev. Anti Infect. Ther..

[15] Mahlapuu M., Hakansson J., Ringstad L., Bjorn C. (2016). Antimicrobial peptides: An emerging category of therapeutic agents. Front. Cell. Infect. Microbiol..

[16] Kang H.K., Kim C., Seo C.H., Park Y. (2017). The therapeutic applications of antimicrobial peptides (amps): A patent review. J. Microbiol..

[17] Akin A., Alp E., Altindiş M., Azak E., Batirel A., Çağ Y., Durmuş G., Kepenek Kurt E., Sağiroğlu P., Türe Z. (2018). Current diagnosis and treatment approach to sepsis. Mediterr. J. Infect. Microbes Antimicrob..

[18] Fleischmann C., Scherag A., Adhikari N.K., Hartog C.S., Tsaganos T., Schlattmann P., Angus D.C., Reinhart K. (2016). International Forum of Acute Care Trialists. Assessment of global incidence and mortality of hospital-treated sepsis. Current estimates and limitations. Am. J. Respir. Crit. Care Med..

[19] Xu C., Guo Z., Zhao C., Zhang X., Wang Z. (2018). Potential mechanism and drug candidates for sepsis-induced acute lung injury. Exp. Ther. Med..

[20] Park B.S., Lee J.O. (2013). Recognition of lipopolysaccharide pattern by tlr4 complexes. Exp. Mol. Med..

[21] Akira S., Uematsu S., Takeuchi O. (2006). Pathogen recognition and innate immunity. Cell.

[22] Hotchkiss R.S., Moldawer L.L., Opal S.M., Reinhart K., Turnbull I.R., Vincent J.L. (2016). Sepsis and septic shock. Nat. Rev. Dis. Prim..

[23] Cohen J., Vincent J.L., Adhikari N.K., Machado F.R., Angus D.C., Calandra T., Jaton K., Giulieri S., Delaloye J., Opal S. (2015). Sepsis: A roadmap for future research. Lancet Infect. Dis..

[24] Jeon D., Jeong M.C., Jacob B., Bang J.K., Kim E.H., Cheong C., Jung I.D., Park Y., Kim Y. (2017). Investigation of cationicity and structure of pseudin-2 analogues for enhanced bacterial selectivity and anti-inflammatory activity. Sci. Rep..

[25] Jiang Z., Vasil A.I., Hale J., Hancock R.E., Vasil M.L., Hodges R.S. (2009). Effects of net charge and the number of positively charged residues on the biological activity of amphipathic alpha-helical cationic antimicrobial peptides. Adv. Exp. Med. Biol..

[26] Lee E., Shin A., Jeong K.W., Jin B., Jnawali H.N., Shin S., Shin S.Y., Kim Y. (2014). Role of phenylalanine and valine10 residues in the antimicrobial activity and cytotoxicity of piscidin-1. PLoS ONE.

[27] Bahar A.A., Ren D. (2013). Antimicrobial peptides. Pharmaceuticals (Basel).

[28] Kim J., Jacob B., Jang M., Kwak C., Lee Y., Son K., Lee S., Jung I.D., Jeong M.S., Kwon S.H. (2019). Development of a novel short 12-meric papiliocin-derived peptide that is effective against gram-negative sepsis. Sci. Rep..

[29] Kawasaki T., Kawai T. (2014). Toll-like receptor signaling pathways. Front. Immunol..

[30] Kim J., Durai P., Jeon D., Jung I.D., Lee S.J., Park Y.M., Kim Y. (2018). Phloretin as a potent natural tlr2/1 inhibitor suppresses tlr2-induced inflammation. Nutrients.

[31] Lee E., Kim J.K., Jeon D., Jeong K.W., Shin A., Kim Y. (2015). Functional roles of aromatic residues and helices of papiliocin in its antimicrobial and anti-inflammatory activities. Sci. Rep..

[32] Bronstein I., Fortin J.J., Voyta J.C., Juo R.R., Edwards B., Olesen C.E., Lijam N., Kricka L.J. (1994). Chemiluminescent reporter gene assays: Sensitive detection of the gus and seap gene products. Biotechniques.

[33] Munford R.S. (2016). Endotoxemia-menace, marker, or mistake?. J. Leukoc. Biol..

[34] Bian Z., Guo Y., Ha B., Zen K., Liu Y. (2012). Regulation of the inflammatory response: Enhancing neutrophil infiltration under chronic inflammatory conditions. J. Immunol..

[35] Bijli K.M., Kanter B.G., Minhajuddin M., Leonard A., Xu L., Fazal F., Rahman A. (2014). Regulation of endothelial cell inflammation and lung polymorphonuclear lymphocyte infiltration by transglutaminase 2. Shock.

[36] Martin T.R. (2002). Neutrophils and lung injury: Getting it right. J. Clin. Investig..

[37] Van der Poll T., van de Veerdonk F.L., Scicluna B.P., Netea M.G. (2017). The immunopathology of sepsis and potential therapeutic targets. Nat. Rev. Immunol..

[38] Wang J., Gong S., Wang F., Niu M., Wei G., He Z., Gu T., Jiang Y., Liu A., Chen P. (2019). Granisetron protects polymicrobial sepsis-induced acute lung injury in mice. Biochem. Biophys. Res. Commun..

[39] Ventola C.L. (2015). The antibiotic resistance crisis: Part 1: Causes and threats. P T.

[40] Diamond G., Beckloff N., Weinberg A., Kisich K.O. (2009). The roles of antimicrobial peptides in innate host defense. Curr. Pharm. Des..

[41] Matsuzaki K. (2009). Control of cell selectivity of antimicrobial peptides. Biochim. Biophys. Acta.

[42] Zelezetsky I., Tossi A. (2006). Alpha-helical antimicrobial peptides--using a sequence template to guide structure-activity relationship studies. Biochim. Biophys. Acta.

[43] Al-Lawama M., Aljbour H., Tanash A., Badran E. (2016). Intravenous colistin in the treatment of multidrug-resistant acinetobacter in neonates. Ann. Clin. Microbiol. Antimicrob..

[44] Lin Z., Zhao X., Huang J., Liu W., Zheng Y., Yang X., Zhang Y., Lamy de la Chapelle M., Fu W. (2019). Rapid screening of colistin-resistant escherichia coli, acinetobacter baumannii and pseudomonas aeruginosa by the use of raman spectroscopy and hierarchical cluster analysis. Analyst.

[45] Jenssen H., Hamill P., Hancock R.E. (2006). Peptide antimicrobial agents. Clin. Microbiol. Rev..

[46] Pal T., Sonnevend A., Galadari S., Conlon J.M. (2005). Design of potent, non-toxic antimicrobial agents based upon the structure of the frog skin peptide, pseudin-2. Regul. Pept..

[47] Tossi A., Sandri L., Giangaspero A. (2000). Amphipathic, alpha-helical antimicrobial peptides. Biopolymers.

[48] Kang H.K., Seo C.H., Luchian T., Park Y. (2018). Pse-t2, an antimicrobial peptide with high-level, broad-spectrum antimicrobial potency and skin biocompatibility against multidrug-resistant pseudomonas aeruginosa infection. Antimicrob. Agents Chemother..

[49] Angus D.C., van der Poll T. (2013). Severe sepsis and septic shock. N. Engl. J. Med..

[50] Martin L., van Meegern A., Doemming S., Schuerholz T. (2015). Antimicrobial peptides in human sepsis. Front. Immunol..

[51] Lu Y.C., Yeh W.C., Ohashi P.S. (2008). Lps/tlr4 signal transduction pathway. Cytokine.

[52] Lee E., Shin A., Kim Y. (2015). Anti-inflammatory activities of cecropin a and its mechanism of action. Arch. Insect Biochem. Physiol..

[53] Cheon D., Kim J., Jeon D., Shin H.C., Kim Y. (2019). Target proteins of phloretin for its anti-inflammatory and antibacterial activities against propionibacterium acnes-induced skin infection. Molecules.

[54] Jnawali H.N., Lee E., Jeong K.W., Shin A., Heo Y.S., Kim Y. (2014). Anti-inflammatory activity of rhamnetin and a model of its binding to c-jun nh2-terminal kinase 1 and p38 mapk. J. Nat. Prod..

[55] Xu W., Huang M., Zhang Y., Li H., Zheng H., Yu L., Chu K., Lin Y., Chen L. (2016). Extracts of bauhinia championii (benth.) benth. Attenuate the in fl ammatory response in a rat model of collagen-induced arthritis. Mol. Med. Rep..

[56] Mushtaq N., Redpath M.B., Luzio J.P., Taylor P.W. (2004). Prevention and cure of systemic escherichia coli k1 infection by modification of the bacterial phenotype. Antimicrob. Agents Chemother..

[57] Kaczmarek A., Budzynska A., Gospodarek E. (2014). Detection of k1 antigen of escherichia coli rods isolated from pregnant women and neonates. Folia Microbiol. (Praha).

[58] Jnawali H.N., Jeon D., Jeong M.C., Lee E., Jin B., Ryoo S., Yoo J., Jung I.D., Lee S.J., Park Y.M. (2016). Antituberculosis activity of a naturally occurring flavonoid, isorhamnetin. J. Nat. Prod..

[59] Park H.J., Lee S.J., Cho J., Gharbi A., Han H.D., Kang T.H., Kim Y., Lee Y., Park W.S., Jung I.D. (2018). Tamarixetin exhibits anti-inflammatory activity and prevents bacterial sepsis by increasing il-10 production. J. Nat. Prod..

[60] Otvos L., Wade J.D. (2014). Current challenges in peptide-based drug discovery. Front. Chem..

